# Feature-rich multiplex lexical networks reveal mental strategies of early language learning

**DOI:** 10.1038/s41598-022-27029-6

**Published:** 2023-01-26

**Authors:** Salvatore Citraro, Michael S. Vitevitch, Massimo Stella, Giulio Rossetti

**Affiliations:** 1 Department of Computer Science, University of Pisa, Largo Bruno Pontecorvo 3, Pisa, Italy; 2 Institute of Information Science and Technologies “A. Faedo” (ISTI), National Research Council (CNR), G. Moruzzi 1, Pisa, Italy; 3 Department of Psychology, University of Kansas, Lawrence, USA; 4 Present Address: Dipartimento di Psicologia e Scienze Cognitive, University of Trento, Rovereto, Italy

**Keywords:** Computer science, Computational science, Human behaviour

## Abstract

Knowledge in the human mind exhibits a dualistic vector/network nature. Modelling words as vectors is key to natural language processing, whereas networks of word associations can map the nature of semantic memory. We reconcile these paradigms—fragmented across linguistics, psychology and computer science—by introducing FEature-Rich MUltiplex LEXical (FERMULEX) networks. This novel framework merges structural similarities in networks and vector features of words, which can be combined or explored independently. Similarities model heterogenous word associations across semantic/syntactic/phonological aspects of knowledge. Words are enriched with multi-dimensional feature embeddings including frequency, age of acquisition, length and polysemy. These aspects enable unprecedented explorations of cognitive knowledge. Through CHILDES data, we use FERMULEX networks to model normative language acquisition by 1000 toddlers between 18 and 30 months. Similarities and embeddings capture word homophily via conformity, which measures assortative mixing via distance and features. Conformity unearths a language kernel of frequent/polysemous/short nouns and verbs key for basic sentence production, supporting recent evidence of children’s syntactic constructs emerging at 30 months. This kernel is invisible to network core-detection and feature-only clustering: It emerges from the dual vector/network nature of words. Our quantitative analysis reveals two key strategies in early word learning. Modelling word acquisition as random walks on FERMULEX topology, we highlight non-uniform filling of communicative developmental inventories (CDIs). Biased random walkers lead to accurate (75%), precise (55%) and partially well-recalled (34%) predictions of early word learning in CDIs, providing quantitative support to previous empirical findings and developmental theories.

## Introduction

The mental lexicon is the part of memory that stores information about a word’s meanings, syntactic features, pronunciation and more^1–3^. Although often described as being like a mental dictionary^1,4,5^, the mental lexicon is not static, and is instead a complex system, whose structure influences language processing and has been investigated across fields like psychology^1^, linguistics^3,6^, computer science and artificial intelligence^7–9^. Decades of multidisciplinary research have gathered evidence that words in the mental lexicon have a dual representation^5^, analogous to the particle/wave duality of light in physics^10^. Psycholinguistics and distributional semantics posit that words in the lexicon possess both a networked organisation^11–13^ and a vector-space nature^14–17^. On the one hand, networks capture conceptual relationships (as links) between words (as nodes). On the other hand, vector-spaces identify alignment and distances between vectors, whose components represent word features. The network aspects of the mental lexicon started with seminal work by Quillian^12^ and by Collins and Loftus^11^. These works showed how in a network of words linked through semantic associations, e.g. possessing a common attribute or overlapping in meaning, the length of the shortest path separating concepts was predictive of retrieval times from semantic memory and sentence understanding^11,12^. The advent of network science has revived interest in this approach^6^, with several recent works examining how the structure of semantic networks^18–22^, phonological networks^13,23^, and their multiplex combination^24–26^ influence language acquisition and processing.

In parallel, distributional semantics postulates that semantic memory possesses a vector space structure^14,15,27^, where concepts are vectors whose components express either interpretable features^28^ (e.g. possessing a semantic feature, being in a category or being acquired at a certain age) or latent aspects of language^16,27,29,30^ (e.g. overlap in meaning due to word co-occurrence in the same context). Although latent aspects of language limit the understanding of cognitive processing, models like Latent Semantic Analysis^16^ and the Hyperspace Analogue to Language^30^ were used extensively in cognitive inquiries of information processing, mainly due to their ability to extract semantic features without human intervention. More recently, transformer neural networks like BERT enabled vector representations for words depending on their context^14^. This enhancement revolutionised the field of natural language processing and predicted successfully semantic tasks like entity recognition or word meaning disambiguation^14,29^. Although powerful predictors, these approaches provide relatively little access to the organisation of words in the human mind and can thus benefit from network models and interpretable distributional semantics^29^. Reconciling the non-latent, interpretable vector/network duality of words in the mental lexicon is the focus of this work.

We introduce FEature-Rich MUltiplex LEXical - *FERMULEX* - networks, a framework combining the vector-based and multiplex network aspects of words and their associations in the mental lexicon. Rather than merely building networks out of similarities between vectors of features^31^, we view structure and feature similarities as two independent building blocks, whose contribution to represent words in the mind can be explored in parallel. Hence in *FERMULEX* networks, network structure remains and can be explored even when word similarities are switched off, and vice versa. This possibility does not exist in networks built from vector similarities (cf.^32^). We achieve this advancement by using the recent measure of conformity^33^, an enhancement of assortative mixing estimation through non-adjacent nodes.

**Figure 1:**
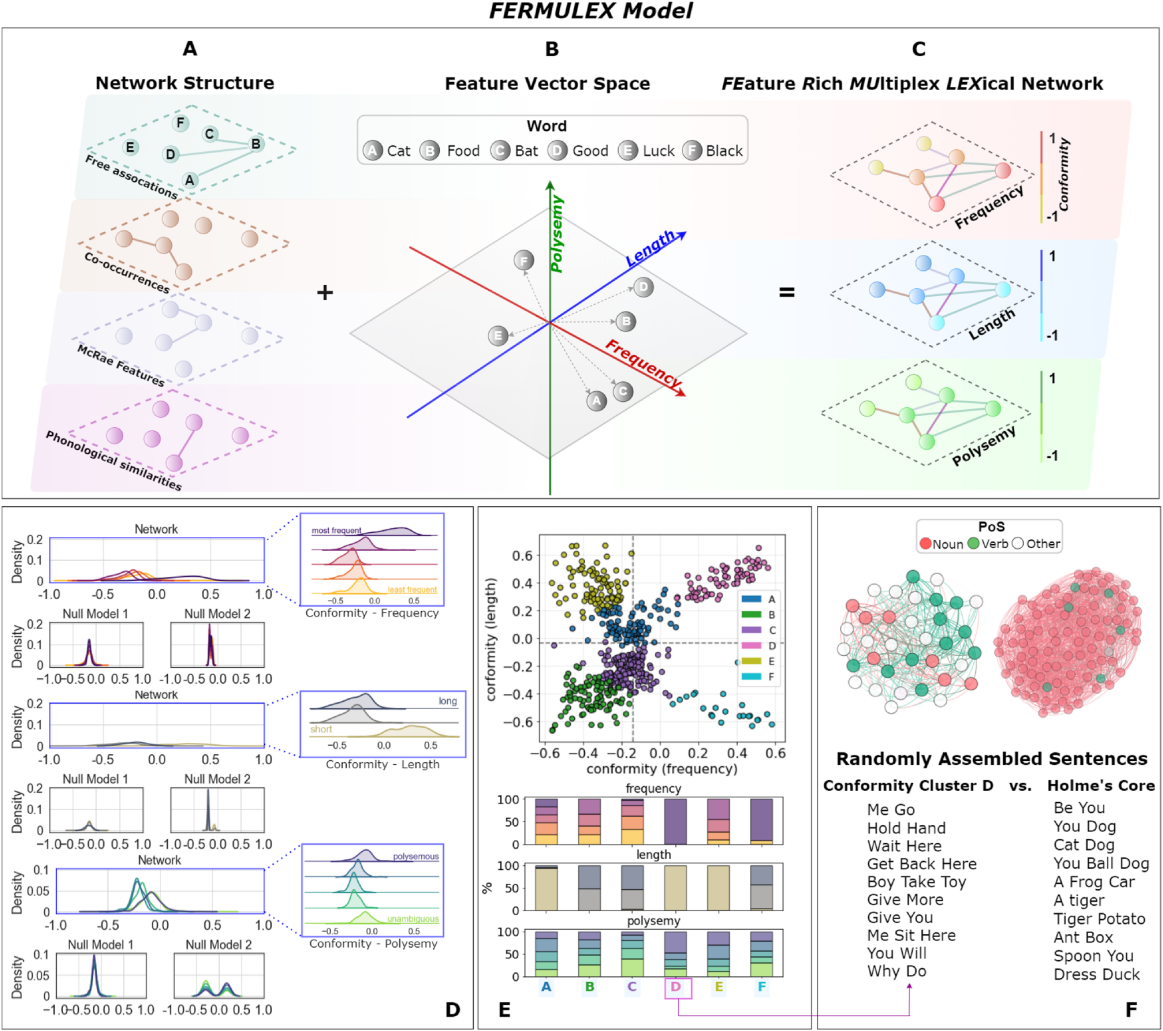
(**A**–**C**) Combining multiplex topology (**A**) and vector spaces (**B**) results in *FERMULEX* network (**C**); (**D**) kernel density estimates (KDEs) and ridgeline plots highlight conformity distribution for the frequency, length, and polysemy features in toddlers’ mental lexicon and the randomised variants; (**E**) Above—two-dimensional scatter plot of conformity vector space, where each point is colored according to the cluster the point belongs to (K-means algorithm); Below—distribution of word features within each cluster, where a kernel language emerges, i.e. the cluster labeled as *D*; (**F**) content characterisation of the kernel compared to a competitor from a k-core decomposition.

As outlined in Fig. 1A–C, *FERMULEX* starts from a given multiplex network structure, where nodes represent concepts/words linked by different types of conceptual associations (Fig. 1A). We focus on layers that were found to predict early word learning in toddlers and consider semantic, syntactic and phonological associations between words (see^7,24^ and “Methods”). Each word is also endowed with a vector of psycholinguistic features, i.e. features of relevance for lexical acquisition, processing and storage^3^. We here endow words with vectors of interpretable features, like frequency, length and polysemy (Fig. 1B). In *FERMULEX*, merging network structure with vectorial similarities means measuring how similar any two nodes/vectors can be according to their vector similarity, weighted through network connectivity. This is quantitatively implemented via conformity^33^, which measures a tendency for nodes/words with similar vectors to be separated by shorter distances (i.e. fewer links). Each node receives its conformity score, leading to a richer (in terms of nodes features) multiplex representation (Fig. 1C) of conceptual knowledge in the mental lexicon.

We show that the dual network/vector representation of words is crucial for understanding key aspects of the mental lexicon that would go undetected by considering features—or networks—only. Using normative word learning norms^34^ and phonological/semantic/syntactic^24^ data in 1000 English toddlers, *FERMULEX* networks reveal a language kernel progressively built in the mental lexicon of toddlers and *undetectable* by either network core detection^35^ or clustering in vector spaces^36^. This mental kernel contains general yet simple nouns and verbs that can build diverse sentences, with crucial relevance to children’s communication^37^. The identification of this kernel via *FERMULEX* provides quantitative evidence and modelling insights as to how can young children produce early sentences, as recently observed^37^.

Modelling word acquisition as increasingly biased random walkers over the network/vectorial *FERMULEX* representation leads to more insights. We adopted this approach inspired by past work using random walkers over cognitive networks for investigating the mental lexicon^38^. We find that predicting word learning in the language kernel crucially depends on: (i) network/vectorial conformity^33^ and (ii) the filling of communicative developmental inventories (CDIs)^39^, i.e. lists of words sharing a semantic category and commonly used for measuring early cognitive development. We find that CDIs display a rich filling dynamic in word learning, which can be predicted by our biased random walkers. The results are statistically significant with respect to a baseline random learner. Without combining structural and attributive information as well as CDI filling levels, in fact, predictions of word learning in the language kernel are equivalent to random guessing. Since the language kernel stores words crucial for producing early sentences, our results indicate that the documented ability for young toddlers to communicate via early sentences around month 30^37^ crucially depends on network, vector, and categorical aspects of the mental lexicon. Our approach with *FERMULEX* can encompass them all and thus represents a powerful tool for future cognitive research of various aspects of language.

## Results

### *FERMULEX* characterisation

A combination of a multiplex network structure (Fig. 1A) and a vector space of interpretable features (Fig. 1B) results in a *FERMULEX* network (Fig. 1C). Conformity^33^ assesses structure-feature relationships on the aggregated topology. For each node and with respect to each feature, conformity quantifies the node assortative mixing, by extending this estimation to the non-adjacent but still reachable neighbors of a node. Studying conformity distributions, we can capture heterogeneous patterns between nodes.

Figure 1D sums up these patterns on the real data representing toddlers’ mental lexicon (see “Methods” for details on network layers and vectors of word features). Conformity with respect to frequency highlights an assortative mixing pattern but limited only to highly frequent words, i.e. only words occurring many times in child-directed speech tend to connect with each other in children’s *FERMULEX* network. This effect is absent in lower-frequency words and it was not detected in single-layer semantic networks of adults^40^. Conformity of word length highlights an assortative mixing pattern of very short words only. These two effects are expected to be related as shorter words tend to be more frequent in language^25^.

Interestingly, conformity quantifies that polysemous words are likely to connect to each other to a smaller extent than most frequent and shortest words. This indicates an organisation of concepts where unambiguous/less polysemous words are linked to ambiguous/more polysemous words. This heterogeneous mixing by polysemy could be beneficial in providing context and differentiating among possible meanings of a polysemous word, as suggested by previous studies^40,41^. If all ambiguous words were grouped together, sense disambiguation could not rely on links including less polysemous/unambiguous words and this homogeneity would ultimately violate the frequency-meaning law^42^.

The above assortative mixing patterns are not a consequence of feature/distance distributions, because reshuffling node labels (*Null Model 1*) and rewiring links (*Null Model 2*) disrupt the heterogeneous mixing behaviour among classes (see “Methods” and SI). Hence, the above patterns indicate the presence of a core-periphery organisation in the dualistic multiplex/feature-rich structure of the mental lexicon: A set of highly frequent/shorter/polysemous words linked with each other creates a network core highlighted by conformity and invisible to previous inquiries^7,24^. This preliminary evidence calls for further analysis of the core.

Figure  1E introduces an analysis of the core performed on: (i) dualistic network/vector and (ii) individual aspects of words in the mental lexicon of toddlers (see “Methods” and SI). We aim to find a language core that contains groups of words sharing similar structure-feature relationships. Among the six optimal clusters found (see “Methods” and SI), groups A and B (blue and gold) contain mostly short words. Cluster F (cyan) contains highly frequent words. Cluster D contains short, highly frequent and a relevant portion of polysemous words. Sets of clustered words with such features are known as language kernels in cognitive network science^18,19,25^. Language kernels facilitate communication through a small set of simple words suitable for expressing ideas in multiple contexts^19^. The conformity core (cluster D) satisfies this definition. In fact, 13% of the core is made of nouns, 33% of verbs and the other 54% include adjectives, adverbs and pronouns, which make it more likely to assemble syntactically well-formed sentences by random sampling compared to other word clusters (cf. Fig. 1F). Identifying a network core via k-core decomposition^35^ shows almost no meaning organisation and more expressions that are syntactically unrelated . See two random samples in Fig. 1F: The conformity cluster can form syntactically coherent trigrams such as “Get Back Here” and “Boy Take Toy”, whereas the same does not happen in the only network-based core. Analogously, K-Modes^43^ attribute-only clusters are unable to form syntactically coherent bigrams. See SI for an analysis centered on computing the internal syntactic coherence of the cores. These comparisons provide unprecedented evidence showing a syntactically advantageous organisation of words in early children’s lexicon. This phenomenon goes undetected unless both the network and vector nature of words in the mind is considered.

### Topology and cognitive relevance of the conformity core *FERMULEX*

**Figure 2:**
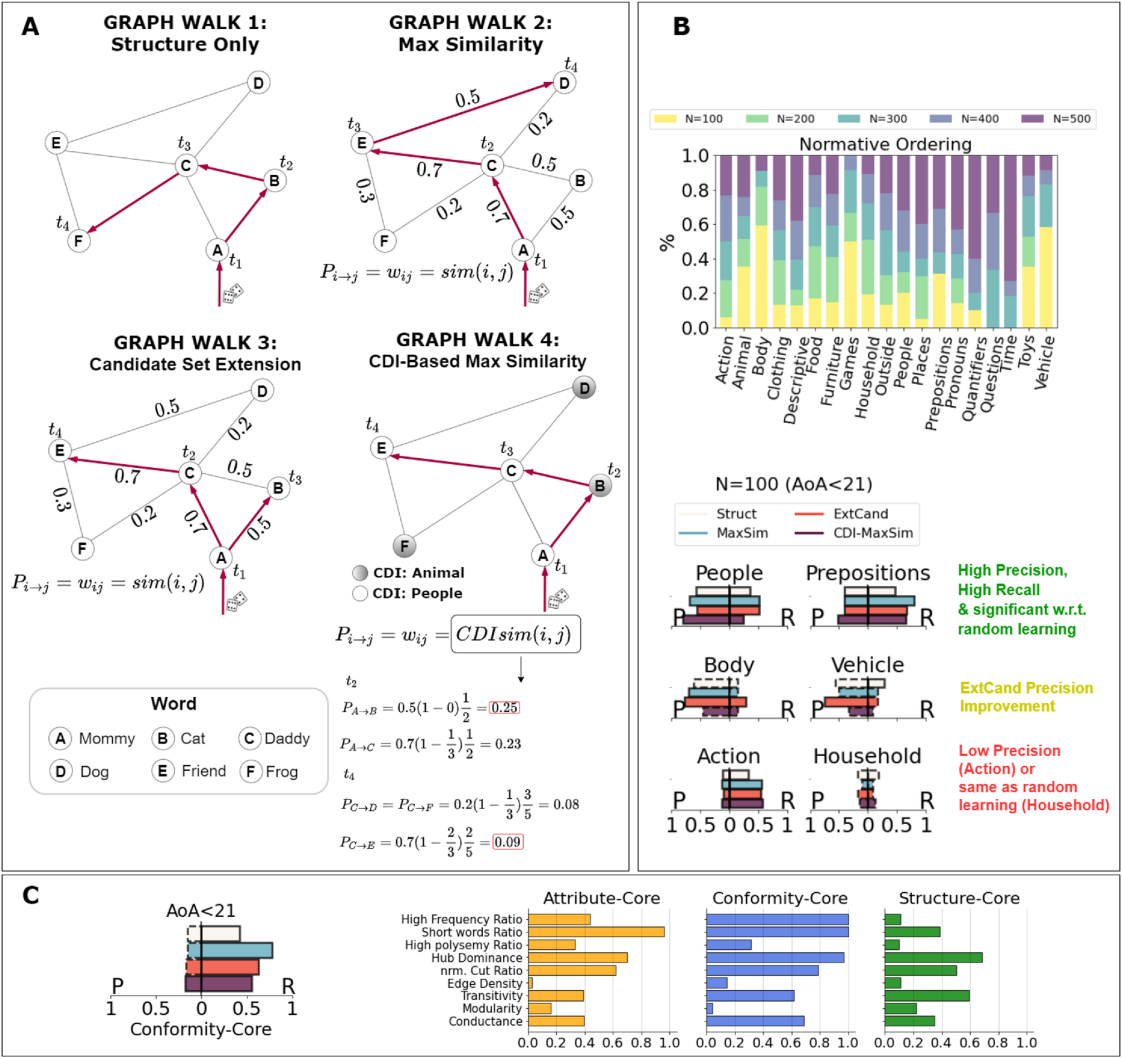
(**A**) Random walks combining progressively structure and vector information (Graph Walk 1–3) and CDIs integration (Graph Walk 4); (**B**) Above—CDIs filling in CHILDES normative learning; bars show that CDIs are not uniformly filled over time, e.g. more than half of *Body* and *Vehicle* categories are learned during early stage acquisition, whereas *Questions* and *Time* emerge later; Below—precision-recall evaluation over selected CDIs; solid bars identify statistically significant scores compared to a random learning baseline; (**C**) Left—precision-recall evaluation with respect to early acquisition of kernel words; Right—kernel characterisations using several quality measures.

We further compare the conformity core with the k-core decomposition^35^ (where similarities are switched off) and with the most relevant K-Modes cluster (where network structure is switched off). Interestingly, the conformity core appears to be a synthesis of the other two potential language kernels. Figure 2C characterises the three cores with several qualitative functions assessing intra-cluster connectivity and inter-cluster distinctiveness (cf. “Methods” and the SI). The K-Modes core contains a rich set of short, highly frequent and polysemous words compared to the conformity core. The conformity core contains a more homogeneous set of words, which is crucial for syntactic sentences mixing specific and more general concepts^19,41,44^. The structural k-core has high transitivity, but the conformity core has a more *cliquish* configuration due to higher hub dominance score^45^. Cliquishness was recently shown to correlate with better recall from memory^46^ due to the concentration of spreading activation in the clique^21^. These recent studies suggest that the higher cliquishness found here for the conformity core might be beneficial for language processing in toddlers. The conformity core also displays high values of conductance and cut ratio: this language kernel possess a dense internal structure but it is also strongly connected to the rest of the graph as well, considerably more than the other competitors. In other words, the conformity core is strongly internally connected (more than k-core) and homogeneous with respect to the features (more than k-mode). This higher connectivity might reflect an advantage in accessing and producing items from the language kernel in view of activation spreading models of the mental lexicon^6,21,22,26^.

### Language kernel entanglement

We aim to further investigate the multiplex structure of the conformity core even through layers. To this aim, we leverage the concept of layer entanglement^47,48^, assessing how much the layers overlap and are balanced in the multiplex core against the whole multiplex structure. In detail, layer entanglement can be captured by two measures^47^: Entanglement intensity *I*, that computes how much layers overlap with other layers, and entanglement homogeneity *H*, that measures how much nodes are connected in a balanced way across layers. In the whole multiplex structure, we find that $I_{tot}=0.09$ and $H_{tot}=0.83$, while in the conformity core/language kernel we find higher intensity and lower homogeneity values, $I_{kernel}=0.29$ and $H_{kernel}=0.64$. A higher entanglement intensity in the kernel ($I_{kernel}>I_{tot}$) demonstrates that such group of words, highlighted by network/vector conformity, acts as a core in the multiplex network: Layers are more entangled, i.e. concentrate more links, within this core rather than in the whole multiplex structure. A lower entanglement homogeneity in the kernel ($H_{kernel}<H_{tot}$) indicates that one or more layers are over-represented in the kernel itself. Looking at the counts of links from different layers with both endpoints within the kernel, we notice that co-occurrences constitute most of the links in the kernel (0.76% of co-occurrences against 0.19% of associations and 0.05% of phonological similarities). This finding provides additional evidence that the observed language kernel is crucial for syntactic relationships, which are best captured by child-directed co-occurrences^49,50^. Interestingly, excluding the layer of co-occurrences from the multiplex network does not alter the presence of the kernel (see SI) nor its entanglement: Entanglement values of the kernel without co-occurrence links do not drastically change, i.e. $I_{partial\_core}=0.39$ and $H_{partial\_core}=0.77$. Moreover, layer entanglement can be computed on a temporal network as well^47^. By creating subgraphs of the original multiplex network with the first 200, 300 and 400 learned words, we registered values of *H* and *I* analogous to the ones of the full multiplex network. Interestingly, the above findings indicate that the language kernel highlighted by the interplay of vector and network features is highly entangled across semantic, phonological and syntactic aspects of the mental lexicon and it persists over time. These patterns further suggest the kernel/core might play a relevant role for supporting cognitive processing (see “Discussion”).

### Normative word learning as random walks on *FERMULEX*

To investigate how the conformity core and the whole *FERMULEX* structure emerge over time, we adopt a random walk framework. Random walks on cognitive network structures have successfully modelled phenomena like Zipf’s law^42^ or semantic priming^38^. Here, we use structure-feature biased random walks to explore normative language learning, as reported in Fig. 2.

The simplest idea is to limit the walk to network structure only (*Graph Walk 1*). To explore the interplay between topology and features of words, we can weigh network links with the similarity between vectors representing adjacent words (*Graph Walk 2*). Let us consider an example. In Fig. 2, at $t_2$, *Graph Walk 1* should choose to learn either *cat* or *daddy* after the current word *mommy*. Because of network/vectorial similarities, *Graph Walk 2* will select *daddy* as the *next-to-be-learned* word. We can expand the set of next-to-be-learned candidate words: *Graph Walk 3* encodes this parallel word learning process by considering as potential candidates all neighbors of already learned words. With reference to Fig. 2A, at $t_3$, *Graph Walk 2* can only move to and learn *friend*, while *Graph Walk 3* can also activate and learn *cat* after *mommy*. Focus is given to considering how these models can predict the assembly over time of: (i) the conformity core, and of (ii) Communicative Development Inventories^39^ (CDIs), which are commonly used by psycholinguists to measure a child’s communicative, receptive and expressive abilities. CDIs are clusters of words from the same semantic category—e.g. a list of words all relative to *time*—and thus represent a portion of the whole vocabulary available to children^51^.

### CDIs are not filled uniformly under normative learning

In the CHILDES data^51^, toddlers are found not to learn CDIs uniformly over time (cf. Fig. 2B). This means that some semantic domains of toddlers’ lexicon are filled earlier during normative learning. However, the above random walkers do not include information about the semantic category a word belongs to. *Graph Walk 4* proposes a CDI-based similarity integrating information about CDIs’ availability and attractiveness. In the figure, at $t_2$, *Graph Walk 4* moves from *mommy* to *cat*, because *Animal*-CDI is relatively emptier than *People*-CDI, i.e. *People* already contains *mommy*. However, at $t_4$, the model learns *friend* from *daddy*, because the *feature similarity equation* term is stronger than the CDI-based ones (see “Methods” and SI).

### Toddler’s language kernel rises from CDI density and network/vector dualities

Figure 2C, left reports precision and recall in reconstructing the conformity core early on during cognitive development. Performance metrics statistically higher than random learning (significance of 0.05, see SIB) are highlighted with full bars. Non-significant results are visualised as dashed bars. The normative growth of children’s language kernel was captured with a precision higher than random learning only by our most advanced model, combining CDI density, multiplex network structure and feature similarities. This provides strong evidence that semantic spheres and their filling over time provide insights additional to network/vector duality for capturing how early production of syntactically coherent sentences is achieved^37^. Compared to other CDIs (see next section), our walkers achieved a relatively lower precision in predicting the assembly of the conformity core. This indicates that the language kernel *does not* emerge all at once during early cognitive development, unlike other kernels highlighted in older children^25^. The emergence of the conformity core is thus a gradual phenomenon, that is not strongly biased by similarities and cannot thus be captured by biased random walks only.

### Random walks highlight different strategies at work in different CDIs

Random walks produce word ordering lists that we evaluate with respect to CHILDES normative ordering, i.e. the order in which most children produced words over time (Fig. 2B-above). Random learning is used as a baseline to test whether walks considering word topology and feature predict more words as correctly learned over time. See “Methods” and SI for details of our statistical approach.

Table 1 presents a coarse-grained evaluation of the walkers (cf. “Discussion”). Figure 2B sums up results with respect to CDIs focusing on the very early stage of acquisition, which corresponds to $N=100$ words learned before 21 months^24^. The selected CDIs are captured differently by the models. CDIs like *People* and *Prepositions* are predicted with higher-than-random precision and recall for all Graph Walk models. *CDI-MaxSim* precision is slightly better than in the other models. Interestingly, the two most filled CDIs in this stage of acquisition, i.e. *Body* and *Vehicle*, are predicted with high precision but low recall (cf. “Methods” and SI). This means that the few words predicted are the expected ones, but the models cannot fill the CDIs. *ExtCand* precision is higher. Not all CDIs can be predicted in this way, e.g. *Action* and *Household*. Furthermore, model performances for *Household* are not distinguishable from a random learning, i.e. all bars are dotted. The high recall but the low precision of *Action* is poorly relevant: less of 0.1% of the CDI is covered in this stage of acquisition (however, cf. the SI, where *Action* category is well captured in other stages).

**Table 1:** Model performances over each bin of acquisition.

	Accuracy	Relevant CDIs	Precision	Relevant CDIs	Recall	Relevant CDIs
**AoA < 21**						
Random learning	0.67	–	0.17	–	0.19	–
Struct	0.70	0.26	0.40	0.64	0.30	0.58
MaxSim	0.76	0.26	0.37	0.70	0.34	0.52
ExtCand	0.65	0.21	0.55	0.76	0.30	0.58
CDI-MaxSim	0.75	0.42	0.25	0.58	0.34	0.47
**< AoA < 23**						
Random learning	0.71	–	0.17	–	0.19	–
Struct	–	0.00	0.24	0.64	0.24	0.71
MaxSim	0.82	0.36	0.28	0.57	0.25	0.64
ExtCand	0.83	0.26	0.24	0.42	0.24	0.50
CDI-MaxSim	0.66	0.10	0.26	0.71	0.26	0.71
**< AoA < 24**						
Random learning	0.69	–	0.17	–	0.19	–
Struct	0.73	0.21	0.19	0.42	0.21	0.52
MaxSim	0.75	0.36	0.17	0.42	0.23	0.42
ExtCand	0.73	0.31	0.20	0.21	0.21	0.52
CDI-MaxSim	0.69	0.21	0.19	0.52	0.23	0.52
**24 < AoA < 26**						
Random learning	0.70	–	0.17	–	0.19	–
Struct	0.73	0.31	0.20	0.44	0.22	0.61
MaxSim	0.72	0.31	0.21	0.38	0.26	0.44
ExtCand	0.71	0.42	0.18	0.33	0.22	0.50
CDI-MaxSim	0.72	0.31	0.23	0.38	0.22	0.44
**AoA > 26**						
Random learning	0.61	–	0.24	–	0.24	–
Struct	0.68	0.78	0.32	0.72	0.36	0.61
MaxSim	0.70	0.84	0.33	0.77	0.35	0.66
ExtCand	0.64	0.52	0.28	0.44	0.29	0.66
CDI-MaxSim	0.79	0.31	0.33	0.77	0.41	0.66

*Relevant CDI fraction* is the ratio of statistically significant precision/recall values against a random learning model.

## Discussion

This work introduces a cutting-edge combination of network^1,11,18^ and vector^15,16^ aspects of knowledge in the human mind, which historically run in parallel when modelling language and its cognitive processes^6^.

Using data from 1000 toddlers between 18 and 30 months from the CHILDES project^51^, our *FERMULEX* network revealed a core of words facilitating word production^44^ and invisible to methods based on network structure^24,25,35^ or vector similarities only. This core was detected via conformity^33^, a metric extending assortative mixing estimation in a multi-scale, node-centric fashion. Our numerical experiments identified this core as a set of highly frequent, short, polysemous and well-connected nouns and verbs, i.e. a language kernel containing concepts versatile enough to communicate via basic sentences (cf.^19^) and whose access via spreading activation is facilitated by network connectivity^6,21,46^. Revealing the presence of such a core through our analyses provides for the first time quantitative support of recent empirical findings showing that typical learners can start combining words in basic sentences after 30 months of age^44^. The kernel persisted even when co-occurrences from child-directed speech were ignored (see SI): the conformity core emerged from an interplay between semantic/phonological associations and psycholinguistic norms in the mental lexicon of linguistic knowledge.

It is important to underline that previous network-only models using the same data^7,24^ were not able to highlight such kernel. Analogously, as shown here, focusing only on vectorial similarities could not identify such kernel either. We thus consider the combination of vectorial and network aspects of associative knowledge to represent an interesting “third direction” of investigation, merging aspects of relevance for investigating how the cognitive reflection of language works. *FERMULEX* inherits from networks the ability to map the local and global layout of associations words engage in, e.g. phonological degree explaining patterns of short-term memory retention^1^ or network distances reproducing patterns of semantic similarity judgements^21^. From vector models of words, i.e. word embeddings^14,52^, *FERMULEX* inherits the ability to encode features of concepts beyond mere network patterns, potentially leading the way to future investigation of distributional semantics^17^ integrating network science within a coherent, mathematical framework.

To investigate the assembly over time of such a crucial core of linguistic knowledge, we implemented artificial models of word learning as biased random walks over *FERMULEX*, inspired by past approaches using walkers to investigate the mental lexicon^38,42^. We found that the conformity core does not emerge suddenly over time, differently from other language kernels modelled as viable component in other studies^25^. Instead, the conformity core is progressively built in ways that are captured only by combining the network and vector aspects of words together with CDI filling rates. This finding quantitatively stresses that the conformity core—containing building blocks for producing syntactically coherent words—emerges from strategies dependent on semantic categories, which are partly captured by CDIs^51^.

We also used the same random walkers for capturing how different CDIs filled over time through normative learning, giving unprecedented focus^24^ to learning strategies for individual aspects of children’s knowledge. In our analyses, different CDIs are found to fill at different times over developmental stages, further emphasizing that language learning is not a uniformly random process. Inventories relative to food and action themes are found to be predicted well by our model, confirming recent independent studies^49,53^ that these salient familiar themes are crucial for predicting early language acquisition.

Notice also that words in some CDIs might be learned according to context-specific strategies^54,55^, so that a single, general word-learning strategy might not fit all cases. For instance, according to the *Pervasiveness Hypothesis* by Clerkin and colleagues^55^, toddlers would tend to learn earlier words more frequently occurring across several daily contexts. This visual prevalence/occurrence would be crucially missing from CDIs like *Household* or *Action*, which were in fact poorly reproduced by our model. These negative findings indicate the presence of local strategies for learning words in physical settings that are at work in toddlers but missing from the current instance of *FERMULEX*.

For inventories like *Body* or *Vehicle*, a combination of network structure and feature similarities corresponded to a significant boost in precision over predictions from random learning. This is quantitative evidence for combining network and vector aspects of the mental lexicon. A further boost in precision was found when the random walker was allowed to backtrack. This indicates that some components of the mental lexicon are not built sequentially, without appending words to the most recent lexical item, as assumed in attachment kernel models^56^, but rather filling gaps in the whole vocabulary available to children, as shown also by other approaches with persistent homology and gap filling^57^.

Interestingly, recency in word acquisition is found to be more a powerful strategy for reconstructing the filling of CDIs like *People* or *Prepositions*, where our most elaborate random walker based on recency beats the back-tracking one. Our quantitative results open the way for further discussion and interpretation in light of psychological studies behind early language learning.

This first conception of *FERMULEX* has some key limitations, which can be addressed in future research. For example, our approach considers only normative learning, i.e. how most children learn words over time^24^. This learning dynamic might be different from how individual children with different language learning skills might learn words over time^8^. Future research should thus test the presence of the language kernel and its time-evolution dynamics in a longitudinal cohort of children. Since the occurrence of the language kernel characterises normative learning in a large population of 1000 and more toddlers^51^ and it supports the production of early sentences observed in normative talkers^37^, we expect for the kernel to be present in normative learners but also to be disrupted or incomplete in late talkers^58^. If supported by data, then the language kernel revealed here could become a crucial early predictor of delayed language development in young children. Another limitation is that our predictions do not treat learning as the outcome of a statistical process, where words are learned with certain probabilities. Rather we model word learning as a binary learned/not learned process. We chose to follow this approach for model parsimony and indicate the addition of statistical learning^59^ within the *FERMULEX* framework as an exciting future research direction. Future enhancements of random-walk models should account also for distinctiveness in addition to similarity. The recent work by Siew^60^ indicates that global feature distinctiveness, i.e. how many different semantic features are possessed by a word, correlates with earlier acquisition. Hence, random walkers accounting for switches between distinctiveness and similarity might enhance prediction results and represent an exciting future research direction. Another important approach for future research might be casting language acquisition as a percolation problem, which has been explored in feature-rich networks only recently^61^. An important limitation of our study is that it adopts CDIs for modelling language learning, however these inventories are not grounded in theories from cognitive psychology^39^ but were rather created *ad-hoc* by psycholinguists. Future instances of *FERMULEX* networks should rely on word learning data that is more representative across semantic and syntactic categories.

## Methods

### Multiplex layers

We modelled word learning as a cognitive process acting on a mental representation of linguistic knowledge. Structure in this representation is given by a multiplex lexical network, where nodes represent words that are replicated and connected across different semantic and phonological levels of the network^24^.

Only layers of relevance for word learning acquisition were considered^24^, namely: (i) free associations, indicating memory recall patterns between words from semantic memory^62^, (ii) co-occurrences in child-directed speech^24,51^, (iii) feature-sharing norms, indicating which concepts shared at least one semantic feature from the McRae dataset^63^ and (iv) phonological similarities^13^, representing which words differed by the addition/substitution/deletion of one phoneme only. Hills and colleagues showed that the words with larger degrees in free association networks were also more likely to be acquired at earlier ages, a phenomenon known also as *lure of the associates* (cf. also^64^). A subsequent study by Carlson and colleagues^65^ found a similar effect also in phonological networks built from child-directed speech^13^. Investigations of co-occurrence and feature sharing networks by Beckage and Colunga reported that highly connected words were distinct trademarks of early word production in typical talkers^9^. Importantly, these four aspects of knowledge in the human mind produced network representations that were irreducible^24^. Layers represented different connectivity patterns among words and could thus not be aggregated or erased without decreasing structural information about the system in terms of Von Neumann graph entropy.

### Normative age of acquisition

Network models of language acquisition often use normative datasets that follow the development of language production in toddlers^64^. The most prominent data source is CHILDES (Child Language Data Exchange System), a multi-language corpus of the TalkBank system established by MacWhinney and Snow, storing data about language acquisition in toddlers between age 16 and 36 months^51^. No new experiments were conducted in the current study, and no new data were generated accordingly. Data were granted to the corresponding author by the TreeBank project after a request from the CHILDES platform (https://childes.talkbank.org/) for secondary analysis. CHILDES and TreeBank have IRB approval and guidelines (https://talkbank.org/share/irb/), so that all researchers joining such research repository, like ourselves, have to abide to these ethical standard in any secondary data analysis, like the current one.

We used CHILDES data to rank words in the order they are learned by most English toddlers. By considering the fraction of children producing a certain word in a given month, within each month, words were assigned a production probability. Month after month, a rank in descending order of production probability was constructed as a proxy for normative learning of most toddlers, as done in previous studies^9,24,66^.

### Features

This study selected word features shown in previous research to influence early language acquisition, namely frequency in child-directed speech^24,51^, word length^8,66^ and polysemy^41^. Polysemy scores indicated the numbers of meanings relative to a given word in WordNet^67^, a proxy to word polysemy successfully used in quantitative studies of early word learning^24^. Due to the highly-skewed distribution of variables (e.g., Zipf’s law for word frequency^68^), we regularised data by recasting it from numerical to categorical, as to avoid biases in computing conformity^33^. We grouped each variable into discrete bins, fine tuning bin boundaries so as to obtain non-empty bins featuring the same order of magnitude of entries. This fine-tuning led to splitting words in quintiles for both word frequency and polysemy and in tertiles for length.

### Conformity

We characterise the interplay between structure and features through conformity^33^, which estimates the mixing patterns of nodes in a feature-rich network, i.e. a categorical node-attributed network. This measure can find heterogeneous behaviour among all nodes of a network. Conformity enables a multi-scale strategy by leveraging node distances for computing label-similarities between a target node and other nodes. A distance damping parameter $\alpha$ is needed for decreasing the impact of label-similarities over longer network distances between the target node and its connected neighbors. Based on previous investigations^33^, we adopt a value of $\alpha =2$ giving more emphasis to closer neighbours in a given network topology. See the SI or^33^ for a formal description of the measure and the motivation behind its choice in this work.

When analysing conformity, we need to test whether the measured values are a trivial consequence of structural (or attributive) patterns or rather come from a non-trivial interplay between the two. To characterise this, we resort to two null models: (i) random re-shuffling the node attribute labels while maintaining network topology (*Null Model 1*, Fig. 1D,^25^), and (ii) randomly rewiring of links while preserving the node degree and attribute labels (*Null Model 2*, Fig. 1D). In other words, let us consider this question: Are two labels at the endpoint of an edge significant for the distribution of conformity or can we observe similar patterns by randomly rewiring the attributive or structural model components? While rewiring labels or connectivity patterns, respectively, we keep the other component fixed. For building *Null Model 1*, a random label permutation is enough to disrupt correlations between structure and features. For building *Null Model 2*, we used a configuration model^69^ to obtain a degree preserving graph randomisation, that is, given *N* nodes and any arbitrary degree sequence $\{k_i\} = (k_1, k_2, k_N)$, we place $k_i$ stubs on each node *i* in the graph; then we match each stub with another one until all stubs are matched. The conformity distributions of the null models in Fig. 1D refer to the average node scores from 100 randomised instances of *FERMULEX* network.

All conformity distributions are analysed through kernel density estimates (KDEs) and ridgelines (Fig. 1D); in particular, these last ones get a better picture of mixing heterogeneity between the class labels on the original toddlers’ lexicon.

### Core: definition and evaluation

For finding a potential language core, we model each word as a vector of conformity scores. This results in a vector space where classic clustering algorithms as K-Means^70^ can be run. We reveal a relevant set of words among the six optimal clusters identified by K-Means through the elbow method. The SI provides methodological details about this configuration.

A set of several quality functions are proposed to characterise the language core. We focus on modularity, conductance, cut ratio, internal edge density, hub dominance and transitivity^71^. Modularity, conductance and cut ratio focus on the links within and outside a community: They measure how well-separated a cluster is from the rest of the network. Edge density, transitivity and hub dominance characterise the internal structure of the core. In particular, transitivity and hub dominance characterise it in terms of triadic closure and *cliquishness* level, i.e. the creation of subgraphs where each node is fully connected to others. See the SI for their formal description. All in all, these network metrics are used to characterise the structure of the different cores found via conformity (in *FERMULEX*), via core-detection on the network structure only^35^ and via K-Modes on feature embeddings only^43^. Notice that these measures, combined, provide info about the distinctiveness and connectedness of a given component/cluster in a network.

### Graph walks

We aim to model early word acquisition by progressively combining the network and vector components of *FERMULEX* to achieve this goal, the core idea is to generate a word rank that is progressively filled according to the different graph walk strategies, each one incorporating specific assumptions. In this work we compare four alternative random walk models each one having a unique rationale on how to weigh links and/or to determine the set of candidates for the next to-be-learned word. In particular:

- *Struct* (Graph Walk 1): Words are connected by unweighted links, hence the next word is chosen according to the underlying structure only. Similarly, the set of candidates is chosen from the adjacent neighborhood of the current word;
- *MaxSim* (Graph Walk 2): Edges are weighted according to the pairwise similarity between nodes’ features. Jaccard similarity is used (cf. SI), and frequency, length and polysemy are all considered. The same strategy of *Struct* is used for the set of candidates;
- *ExtCand* (Graph Walk 3): The same strategy of *MaxSim* is used for weighing links; the set of candidates is chosen from the adjacent neighborhood of all the words already learned;
- *CDI-MaxSim* (Graph Walk 4): Links are weighted according to a CDI-based pairwise similarity between the attributes of nodes as well as the availability and attractiveness (cf. SI), and it needs to be updated at each iteration. The same strategy of *Struct* and *MaxSim* is used for the set of candidates.

*Struct* and *MaxSim* are biased random walks considering, respectively, topology or similarity between words (i.e., the network structure or the vector space) while *ExtCand* and *CDI-MaxSim* aim for a more holistic approach.

*ExtCand* visit strategy is designed to mime non-sequential word learning in children (cf.^8^), where the word acquired at step $t+1$ could be similar to any word already learned before, thus enabling an interplay between exploration and exploitation of CDIs. When the last word determines the topology of similar candidates for the next acquisition step, resembling a Markovian process^66^, the walker possesses a bias to remain within the same CDI. By considering as to-be-learned candidates all previously learned words, the walker has a chance of backtracking and acquiring more words within the CDI sharing tightly similar concepts.

*CDI-MaxSim*, the CDI-based model relies on pairwise similarity between two words modulated by additional information on the filling of CDIs they belong to. For additional details and a formal description of the pairwise similarity function adopted refer to the SI.

### Graph walk evaluation

Accuracy, precision and recall are used to evaluate the goodness of ranks’ prediction, as commonly done in statistics and machine learning. Accuracy is defined as the number of correct predictions, i.e. true positives or TP, divided by the total number of predictions. In this domain, TPs are words belonging to a CDI that are learned by a random walker in a specific bin of age of acquisition. Precision is the fraction of relevant elements among all the retrieved ones including non-relevant elements, i.e. false positives or FP. In this domain, FPs are words that fill a CDI as expected in a particular age of acquisition bin, but they are not the exact same words considered in normative learning. For instance, *dog* might contribute to increase FPs because it belongs to the *Animals* CDI but the normative learning contemplated *cat* instead of *dog*. Finally, recall is the fraction of relevant elements that are retrieved. Missing relevant elements (false negatives or FNs) are CDI’s words that are not retrieved by a random walker in a particular bin of age of acquisition. The above definitions imply that there can be predictions with high recall and low precision, because there are many words that satisfy the semantic category roughly represented by the CDI (e.g. guessing as learned names of animals) but different from the specific words learned during normative acquisition (e.g. other names of animals). This interplay spans from the specific characterisation of random-walk predictions and it is accounted for in the “Results” and “Discussion” sections. See the SI for a complete formalization of the measures, and toy examples.

## Supplementary Information

Supplementary Information 1.

Supplementary Information 2.

## Data Availability

All the network layers used for this study were obtained from^24^. All word kernels generated during this study are included in this published article and its supplementary information files.

## Code availability

Conformity code for genrating the word kernels is available on https://github.com/GiulioRossetti/conformity.
