# Supplementary material for: Feature-rich multiplex lexical networks reveal mental strategies of early language learning
Source: Sci Rep. 2023 Jan 26;13:1474. doi: 10.1038/s41598-022-27029-6 (PMC9879964; doi:10.1038/s41598-022-27029-6)
Supplement: Supplementary file 2 — Supplementary Information 2. [file 41598_2022_27029_MOESM2_ESM.pdf]

Supporting Information for ”Feature-rich multiplex lexical networks reveal mental strategies of early language learning”

Salvatore Citraro [1,2], Michael S. Vitevitch [3], Massimo Stella [4+], Giulio Rossetti [2+]

1 Department of Computer Science, University of Pisa Largo Bruno Pontecorvo, 3, Pisa
2 KDD-Lab, ISTI (CNR) G. Moruzzi, 1, Pisa
3 Department of Psychology, University of Kansas, USA
4 CogNosco Lab, Department of Computer Science, University of Exeter, UK

Corresponding author: m.stella AT exeter.ac.uk

+ **These authors contributed equally.**

1 Layers

We add here other details about the definition and construction of the four layers, each of them encoding different levels of language among semantic, syntactic, and phonological aspects. The association and McRae feature norm layers provide information about word meanings. The phonological layer is based on similarity patterns across word pronunciations. The layer of co-occurrences in child directed speech likely contains information related to semantic, syntactic, and phonological similarity.

The free association layer is based on the empirical University of South Florida Free Association Norms [12]. The dataset was built over almost 750.000 empirical free association pairs produced by 6.000 participants as responses to 5.019 stimulus words. Participants were asked to indicate the first target word that came to mind which was related to the presented cue word. Thus, it is possible to connect a cue word to its response with a given normalized frequency. In this investigation of multiplex lexical networks, we ignored edge weights and converted links from directed to undirected, thus building a non-weighted and undirected network layer. The feature norms layer is based on the McRae feature norms dataset [10], i.e. a set of feature norms collected from approximately 725 participants for 541 living (e.g. dog) and non-living (e.g. chair) basic (noun) concepts. Participants were asked to list semantic features of each concept, capturing the most salient and most relevant features. In the semantic features layer generated from this feature norming study, words A and B are connected if they share at least $X = 1$ semantic features. For the phonological layer, we computed similarities based on the IPA phonological word transcriptions obtained from WordNet 3.0 [11]. An edge in the phonological layer between words A and B means that the IPA phonological transcriptions of these words have edit distance one. Finally, the co-occurrence layer is based on the CHILDES dataset [8], and it considers word co-occurrences based on child directed speech. In the co-occurrence layer an edge exists between word A and B if word A occurs within five words before or after word B. Because of the high rate of spurious connections, the co-occurrence between two words must have a frequency higher than a given threshold C to be considered as an edge in our graph. We set $C = 45$ in order for the co-occurrence layer to display a connectivity as close as possible to that of the association and feature norms layers.

2 Conformity

2.1 Definition

Conformity provides a multi-scale strategy to estimate local homophily in complex networks, overcoming classic measures as Newman's assortativity [13] that only produce a global, averaged score. Conformity is not the only multi-scale strategy in the literature. Some valuable variants of local Newman's assortativity also exist [14]. The reason behind the choice of conformity rather than other measures is because conformity provides node similarities grounded in the real distances between nodes. Other approaches, for instance, only leverage random walks as a proxy of information about paths of all possible lengths [14, 1].

To the best of our knowledge, no other works have used node-centric homophily estimation in a mental lexicon. These measurements were typically applied in social network analysis [9] or mobility data [2].

In the following, we report a concise description of conformity [16]. Given a node-attributed graph $G = (V, E, A)$, where V is the set of nodes, E the set of edges, and A the set of categorical node attributes, we calculate for a node $u \in V$ the conformity score $\psi(u, \alpha)$ with respect to an attribute $l \in A$. The damping parameter α allows to decrease the impact of label-similarities over longer network distances between the target node and all the reachable neighbors. To define conformity formally, we need a couple of support functions, namely the indicator $I_{u,v}$

$$I_{u,v} = \begin{cases} 1 & \text{if } l_u = l_v \\ -1 & \text{otherwise,} \end{cases} \tag{1}$$

that compares the attribute values of two nodes, and the similarity function f_{u,l_u}

$$f_{u,l_u} = \frac{|\{v | v \in \Gamma_u \wedge l_u = l_v\}|}{|\Gamma_u|}, \tag{2}$$

that computes the ratio of u's first-order neighbors that share the same attribute value l_u. Thus, given a real number α in $[0, +\infty)$, conformity of node $u \in V$ is defined as in the following:

$$\psi(u, \alpha) = \frac{\sum_{d \in D} \frac{\sum_{v \in N_{u,d}} I_{u,v} f_{v,l_v}}{|N_{u,d}| d^{\alpha}}}{\sum_{d \in D} d^{-\alpha}}, \tag{3}$$

where $D = max(\{dist(i, j) | i, j \in V\})$, i.e. the maximum distance among all node pairs. The computed score is normalized to ensure that conformity lies in the range $[-1, 1]$.

2.2 Comments on null models

Some class labels exhibit more assortative mixing than others, e.g. shortest words in conformity by length or most frequent words in conformity by frequency (cf. Results). While reshuffling node labels or rewiring links, we intend to observe whether similar distributions can emerge trivially from random label permutations or random link configurations. The heterogeneous distributions do not emerge while measuring conformity on the ensemble of networks obtained from the two randomisation processes (cf. Results and Methods). In conformity by frequency and by length, null models distributions are mainly disassortative among all classes; hence, the *anomalous* behaviour of most frequent and shortest words is flattened by both randomisation processes. Conformity distribution with respect to polysemy slightly differs from the other two attributes. In particular, the null model that rewires links shows a bi-modal distribution. The explanation behind this behaviour could be similar to the explanation used to describe the quasi uniformly mixed pattern of polysemous words (cf. Results): ambiguous and unambiguous words must link in non-trivial patterns that are harder to break while randomising node connectivity.

2.3 Conformity vector space

Multi-dimensional conformity information is used in the language core analysis for finding a relevant set of words in language acquisition. We model each node as its vector of conformity scores, where conformity by frequency, by

length and by polysemy are the vector components. This allows to build a vector space where classic clustering analysis can be performed. The difference between a clustering method on the features only (cf. K-Modes [5], Results) is that using vector of conformity scores we integrate structure-feature relationships, thus we aim to group words having similar mixing patterns across the features.

An optimal K-Means [7] instance is used to cluster words. For selecting the optimal number of clusters k, we leverage the elbow method, namely plotting the sum of squared errors in function of k and determining the point of inflection of the curve. $k = 6$ is identified as the optimal point and chosen as the number of centroids to initialize the algorithm.

3 Core Evaluation

We report here a description of the quality functions used for characterising the language core(s). All the measures are implemented in the CDLib library [17], and other detailed information can be found in the library documentation. Let $G = (V, E)$ be a graph with $v \in V$ and $e \in E$, and C a partition of G with $c \in C$, with c composed by a subset of V and a subset of E. We aim to characterise a cluster/community/core c with the following measures:

- *Conductance*: the fraction of total edge volume that points outside the community:

$$conductance(c) = \frac{|c|}{2|e_c| + |c|},$$

  where $|c|$ is the cardinality of the community, namely the number of community nodes, and e_c the number of community edges;

- *Edge density*: the internal density of the community set:

$$density(c) = \frac{|e_c|}{\frac{|c|(|c|-1)}{2}};$$

- *Hub dominance*: indicates the ratio of the degree of the most connected node in a community with respect to the theoretically maximal degree within the community, namely

$$Hub\_dom(c) = \begin{cases} 1 & \text{iff} |c| = 1 \\ \frac{max_{v \in c} k_v}{|c|-1} & \text{otherwise,} \end{cases},$$

  where k_v is the degree of node v;

- *Modularity*: measures the strength of the division of a network into sets of well-separated clusters or modules, and it is calculated as the sum of the differences between the fraction of edges that actually fall within a given community and the expected fraction if edges were randomly distributed:

$$Q = \frac{1}{(2e_c)} \sum_{vw} \left[A_{vw} - \frac{k_v k_w}{(2e)} \right] \delta\left(c_v, c_w\right),$$

  where $A_{v,w}$ is the entry of the adjacency matrix for $v, w \in V$, k_v, k_w the degree of v, w and $\delta\left(c_v, c_w\right)$ is an indicator function taking value 1 iff v, w belong to the same community, 0 otherwise;

- *Normalized cut ratio*: is the fraction of existing edges (out of all possible edges) leaving the community:

$$cut\_ratio(c) = \frac{|c|}{2|e_c| + |c|} + \frac{|c|}{2(|e| - |e_c|) + |c|};$$

- *Transitivity*: is the average clustering coefficient of community nodes with respect to their connection within the community itself:

$$CC(c) = \frac{1}{|c|} \sum_{v \in c} \frac{2\Delta}{k_v(k_v - 1)}$$

  where Δ is the number of triangles including node v in the community c.

4 Core Analysis

4.1 Persistence without layers

A key result of this work is the identification of a language kernel with interesting structural properties and non-trivial content organisation. Randomly picked pairs/triads of words from this kernel can build simple, syntactically well-formed sentences (cf. Results). However, it can be observed that this core can appear just because there is the co-occurrence layer in the multiplex network, i.e. links between concepts co-occurring in child-directed speech. A strength of the *FERMULEX* model is the possibility to *switch off* a layer from the structural component. Removing completely a layer also allows us to observe the emergence of an interesting conformity core. Fig. 1 establishes that a language core emerges even when the 1000 co-occurrences links from the child-directed speech layer are removed. The language kernel (here, the cluster labeled as D) persists to show homogeneity across all the features. This gives more strength to the hypothesis that the kernel found with conformity stems from a more broad interplay between semantic and phonological layers. Finally, for a complete overview of the whole clustering result, the few differences we can notice without the co-occurrence layer is a more homogeneous distribution of word length within the clusters (cf. Fig 1, E), probably due to the removal of long words from the network.

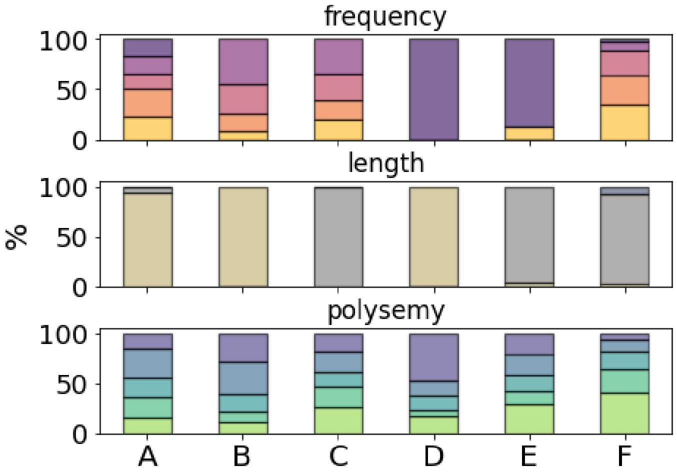

Figure 1: Conformity vector space characterisation without the co-occurrence layer.

4.2 Degree assortativity and hierarchical organisation

We may want to observe whether the removal of the conformity-core can disrupt global characteristics of the network. Several studies [6, 18, 15] identify complex global properties in the degree-degree assortativity and in the hierarchical organisation of lexical networks, measured through $Knn(k)$ and $C(k)$ curves, respectively. $Knn(k)$ curves show the average degree of neighbors of nodes with degree k [6]. Similarly, $C(k)$ curves show the average clustering coefficient of nodes with degree k [15]. If $Knn(k)$ increases with k, the network behaviour is assortative by degree; if $Knn(k)$ decreases with k, the network behaviour is disassortative. If $C(k)$ decreases with k, the network exhibits a hierarchical organisation, otherwise the network does not present this characteristic. Fig. 2 shows that $Knn(k)$ and $C(k)$ curves drastically change when removing the language kernel found with conformity. The network is highly disassortative and hierarchically organised when all nodes are present, but switches to a highly degree-assortative behaviour and splits into two $C(k)$ branches when the core is removed ($r_{Knn} = -0.27$ with $N = V$, $r_{Knn} = 0.55$ with $N = V - V_{core}$; $r_C = -0.33$ with $N = V$, $r_C = 0.32$ with $N = V - V_{core}$). Both disassortativity and hierarchies may spawn from super-general concepts being embedded in the core. These nodes may attract many more links than other nodes, forming a hub-node structure that creates degree disassortativity and implies that the neighbors of hubs are not linked to each other. We suggest that these findings are coherent

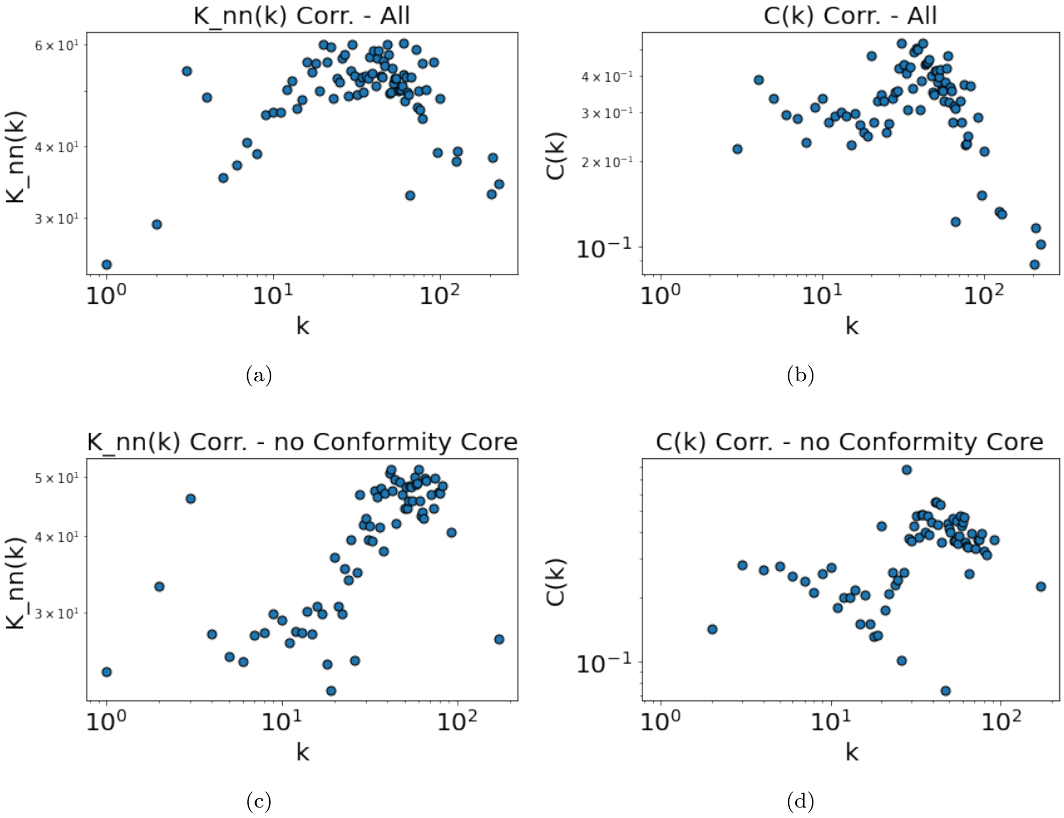

Figure 2: Degree-degree assortativity and hierarchical organisation in CHILDES mental lexicon studying $Knn(k)$ and $C(k)$ curves, respectively, (a-b) with and (c-d) without the conformity core.

with the intra-cluster and inter-clusters analysis to characterise the core (cf. Results). The core is internally structured as a tight *clique*, i.e. it presents high transitivity and hub dominance values. Nevertheless, high conductance/cut-ratio values indicate that the core is highly connected to the rest of the graph. Thus, removing this set of words disrupts the global disassortative and hierarchical organisation of the system, i.e. the underlying structure that guarantees the system connectivity. Further analyses are needed if we aim to interpret these results from a cognitive perspective. Despite some relevant exceptions [19], the lack of a compact set of studies on the degree-disassortative behaviour and the hierarchical organisation of lexical networks makes it difficult to shed light on the underlying cognitive phenomena structuring these non-trivial topological patterns.

4.3 Comparison with other cores

A language kernel is defined as relatively small set of words enabling the creation of simple yet general and frequently used sentences, thus facilitating early communication [3]. This kernel was identified in the cluster found via conformity. Words in this kernel form simple and syntactically structured sentences, and are heterogeneous in part of speech composition. Conversely, the kernel found via k-core network decomposition [4] is unable to form syntactically coherent bigrams/trigrams (cf. Results, Fig. 1, F). For a more robust investigation, we report additional analysis here, including in the discussion the kernel found via K-Modes clustering as well [5].

We focus on the whole internal content organisation of the three cores, regardless of their underlying structure, that we characterized up to now. We aim to compute the ratios of the internal syntactic coherence for the cores. From each core we extract all the possible bigrams. This approach considers each link from the complete subgraph made of core-words, and allows us to count the frequency of each part of a speech pair. We find that

Algorithm 1 Graph Walk

```
Require: Undirected Graph G = (V, E), starting node n
 1: Initialize weights on E
 2: Initialize word ordering list T
 3: Let n be the current visited word and add it to T
 4: Initialize set of word candidates C
 5: while len(T) < |V| do
 6:     if C is not empty then
 7:         for each candidate c in C do
 8:             Compute the similarity between n and c
 9:         Let max(c) be the current word
10:         if max(c) not in T then
11:             add c to T
12:             remove c from C
13:     else
14:         add randomly a not already learned v ∈ V to C
15: return T
```

the most frequent bigram in the structural-based and attribute-based cores is the *noun-noun* pair, 0.42% and 0.20%, respectively. Conversely, the complete subgraph from the conformity-core continues to present a more heterogeneous part of speech composition, where a prominent pair is not observed. In fact, similar frequencies are found for the *verb-noun* (0.1%), the *noun-adjective* (0.06%) and the *verb-adjective* (0.05%) pairs, which are the three most frequent bigrams in the conformity-core.

5 Graph Walks

5.1 Pseudo-code

Algorithm 1 introduces a general schema of a graph walk to describe the four proposed variants. We impose an undirected graph as input, initializing the edge weights as desired (line 1), e.g. all weights are equal to 1 if we want to ignore feature similarity. Then, the word acquisition ordering is initialized, and a randomly selected node n is added to the rank at $t = 1$ (lines 2-3). The set of word candidates is initialized (line 4) to be filled according to the different strategies of each graph walk. Once having a starting word and the first set of candidates, the walk starts until the whole dataset is covered, e.g. each node has a position assigned in the rank (line 5). We iteratively select the new current word by computing the similarity between the current word and the word candidates, choosing the one which maximises similarity (lines 7-9); we re-compute similarity at each iteration because we might need to update this quantity, e.g. when this last one is based on CDI's availability. The new current word is added to the rank only if it was not already learned, otherwise it is removed from the set of candidate (lines 10-12). If the set is empty (line 6), a randomly chosen and still not learned word is added to it for continuing the walk (lines 13-14); this is equivalent to adding an error ϵ, only when it is strictly necessary.

5.1.1 CDI-based similarity

The CDI-based model relies on pairwise similarity between two words i and j modulated by additional information on the CDIs they belong to, namely $i \in I$ and $j \in J$. Let A be the set of features relative to i and B the features of j. The probability for the random walker to acquire j after i is the factorisation of three terms:

$$P_{i \to j} = \begin{cases} J(i,j) & \text{iff} g(cdi(j)) \cdot h(i, cdi(j)) = 0 \\ J(i,j) \cdot g(j) \cdot h(i, cdi(j)) & \text{otherwise,} \end{cases}$$

The first term in $P_{i \to j}$ accounts for the similarity between sets A and B quantified via the Jaccard index, namely $sim(A,B) = |A \cap B| / |A \cup B|$ or the ratio of elements common to both sets of features A and B. The

second term is the target CDI availability, namely the amount of words still available for acquisition in the CDI containing target word j:

$$g(j) = 1 - \frac{|\{w \in J | isactive\}|}{|J|}.$$

The more words are available for acquisition in the target CDI J, the higher $g(j)$ and thus the probability for the random walker to move to $j \in J$. The third term is the CDI attractiveness:

$$h(i, J) = \frac{|\{j \in \Gamma_i | j \in J\}|}{|\Gamma_i|},$$

where Γ_i is the set of adjacent neighbors of the word available for acquisition i. The more i's neighbors are within the target CDI J, the higher $h(i, J)$ and thus the probability for the next word to be *attracted* in the CDI where many neighbors are already present.

5.2 Evaluation

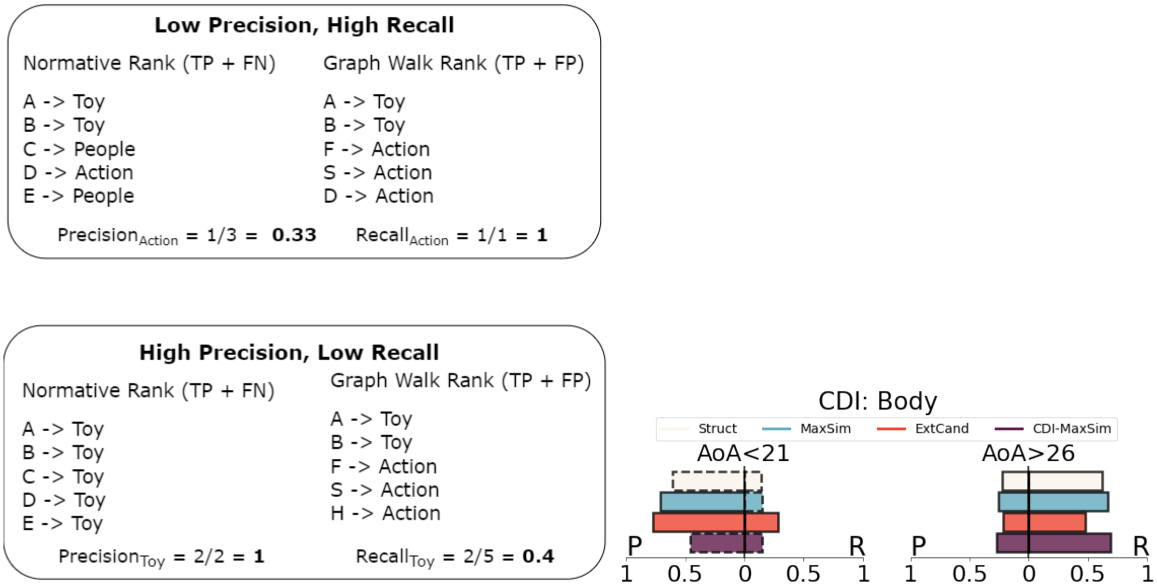

(a) (b)

Figure 3: (a): Toy example focusing on the meaning of precision and recall of a CDI; (b): Precision and recall of *Body* CDI at two different stages of acquisition, i.e., AoA¡21 months, namely the first 100 learned words (N=100), and AoA¿26, namely the last 129 learned words according to CHILDES dataset.

Accuracy, precision and recall evaluate the performances of the random walks (cf. Results and Methods). The measures are built upon the confusion matrix of predictions, i.e. a matrix containing the number of correct predictions, namely true positives (TPs) and true negatives (TNs), and the number of incorrect predictions, namely false positives (FPs) and false negatives (FNs). Contextualizing these concepts in this domain, TPs are CDI's words correctly learned by a random walker in a selected AoA bin, while TNs are all other words that a graph walk correctly predict as not belonging to a CDI in that AoA bin; FPs are words that fill a CDI as expected, but they are not the exact same words considered in normative learning, while FNs are CDI's words that are not retrieved in that AoA bin.

Formally, accuracy is the number of TPs divided by the total number of predictions:

$$Accuracy(CDI, AoA) = \frac{TP}{TP+TN+FP+FN}.$$

Accuracy can answer poorly to some questions as *how many (expected) CDI's words a graph walk can retrieve in a specific AoA bin?* Precision and recall address this better. Hence, precision is the fraction of relevant elements among the retrieved ones,

$$Precision(CDI, AoA) = \frac{TP}{TP+FP},$$

while recall is the fraction of relevant elements that are retrieved,

$$Recall(CDI, AoA) = \frac{TP}{TP+FN}.$$

Example n. 1. We aim to evaluate the performances of a random walk focusing on how the model is filling the *Animal*-CDI at a very early stage of acquisition, i.e. considering the words learned before 21 months. $Recall(Animal, < 21m)$ increases whatever animal-related word the model retrieves, e.g. *dog* and *frog*, but $Precision(Animal, < 21m)$ does not increase if *frog* is not learned before 21 months. FPs as *frog* are non relevant words; moreover, the model can miss FNs as *cat*, i.e. relevant words learned before 21 months.
Example n. 2. Fig. 3 (a) focuses on two possible extremes, i.e. when precision is low but recall is high (above), or precision is high but recall is low (below). In the first case, the normative learning contains only one *Action* word in the sliced AoA bin of five words, but the graph walk retrieves three *Action* words. Recall is maximised, i.e. the expected word D is retrieved; however, FPs as F and S decrease precision. In the second case, *Toy* words only fill the sliced normative bin, but the graph walk correctly predicts two expected words out of five. Precision is maximised, i.e. A and B are expected words; however, FNs as C, D and E decrease recall.

Fig. 3 (b) sums up a real example on the CHILDES dataset. Fig. 4 reports the precision-recall bars of the conformity-core for each bin of age of acquisition (cf. Results, focus on the core words learned before 21 months only). Fig. 5 reports the complete precision-recall bars of each CDI and age of acquisition (cf. Results, focus on *Action*, *Body*, *Household*, *People*, *Prepositions* and *Vehicle* CDIs only, acquired before 21 months).

5.2.1 Random learning

We compare the graph walk performances against a learning model that assigns to all words a position in the rank randomly, regardless of any type of structural, vectorial or CDI-based information (cf. Results). In each precision-recall plot, solid bars specify whether the values are statistically significant with respect to this random word assignment. We use the following z-score for the test:

$$z = \frac{M_1 - M_2}{\sqrt{\frac{\sigma_1^2}{n} + \frac{\sigma_2^2}{n}}}$$

where M_1 is the mean precision/recall value from n runs of the walk model, with σ_1 standard deviations, and M_2 is the mean precision/recall value from n runs of the random learning model, with σ_2 standard deviations. Thus, dotted bars indicate values that are not statistically different from the random learning distribution ($z > 0.05$ or precision/recall higher in the random learning than in the random walk model).

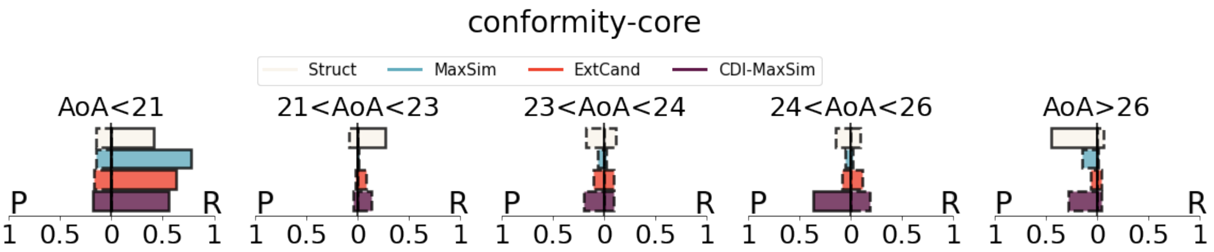

Figure 4: Precision-recall evaluation of the core over all bins of age of acquisition.

Acknowledgments

This work is supported by the European Union – Horizon 2020 Program under the scheme "INFRAIA-01-2018-2019 – Integrating Activities for Advanced Communities", Grant Agreement n.871042, "SoBigData++: European Integrated Infrastructure for Social Mining and Big Data Analytics" (http://www.sobigdata.eu).

References

[1] Aleix Bassolas and Vincenzo Nicosia. "First-passage times to quantify and compare structural correlations and heterogeneity in complex systems". In: *Communications Physics* 4.1 (2021), pp. 1–14.

[2] Eszter Bokányi et al. "Universal patterns of long-distance commuting and social assortativity in cities". In: *Scientific reports* 11.1 (2021), pp. 1–10.

[3] Ramon Ferrer I Cancho and Richard V Solé. "The small world of human language". In: *Proceedings of the Royal Society of London. Series B: Biological Sciences* 268.1482 (2001), pp. 2261–2265.

[4] Petter Holme. "Core-periphery organization of complex networks". In: *Physical Review E* 72.4 (2005), p. 046111.

[5] Zhexue Huang. "Clustering large data sets with mixed numeric and categorical values". In: *Proceedings of the 1st pacific-asia conference on knowledge discovery and data mining,(PAKDD)*. Citeseer. 1997, pp. 21–34.

[6] HaiTao Liu. "Statistical properties of Chinese semantic networks". In: *Chinese Science Bulletin* 54.16 (2009), pp. 2781–2785.

[7] James MacQueen et al. "Some methods for classification and analysis of multivariate observations". In: *Proceedings of the fifth Berkeley symposium on mathematical statistics and probability*. Vol. 1. 14. Oakland, CA, USA. 1967, pp. 281–297.

[8] Brian MacWhinney. *The CHILDES project: The database*. Vol. 2. Psychology Press, 2000.

[9] Miller McPherson, Lynn Smith-Lovin, and James M Cook. "Birds of a feather: Homophily in social networks". In: *Annual review of sociology* (2001).

[10] Ken McRae et al. "Semantic feature production norms for a large set of living and nonliving things". In: *Behavior research methods* 37.4 (2005), pp. 547–559.

[11] George A Miller. *WordNet: An electronic lexical database*. MIT press, 1998.

[12] Douglas L Nelson, Cathy L McEvoy, and Thomas A Schreiber. "The University of South Florida free association, rhyme, and word fragment norms". In: *Behavior Research Methods, Instruments, & Computers* 36.3 (2004), pp. 402–407.

[13] Mark EJ Newman. "Mixing patterns in networks". In: *Physical review E* 67.2 (2003), p. 026126.

[14] Leto Peel, Jean-Charles Delvenne, and Renaud Lambiotte. "Multiscale mixing patterns in networks". In: *Proceedings of the National Academy of Sciences* 115.16 (2018), pp. 4057–4062.

[15] Erzsébet Ravasz and Albert-László Barabási. "Hierarchical organization in complex networks". In: *Physical review E* 67.2 (2003), p. 026112.

[16] Giulio Rossetti, Salvatore Citraro, and Letizia Milli. "Conformity: A path-aware homophily measure for node-attributed networks". In: *IEEE Intelligent Systems* 36.1 (2021), pp. 25–34.

[17] Giulio Rossetti, Letizia Milli, and Rémy Cazabet. "CDLIB: a python library to extract, compare and evaluate communities from complex networks". In: *Applied Network Science* 4.1 (2019), pp. 1–26.

[18] Akira Utsumi. "A complex network approach to distributional semantic models". In: *PloS one* 10.8 (2015), e0136277.

[19] Bram Van Rensbergen, Gert Storms, and Simon De Deyne. "Examining assortativity in the mental lexicon: Evidence from word associations". In: *Psychonomic bulletin & review* 22.6 (2015), pp. 1717–1724.

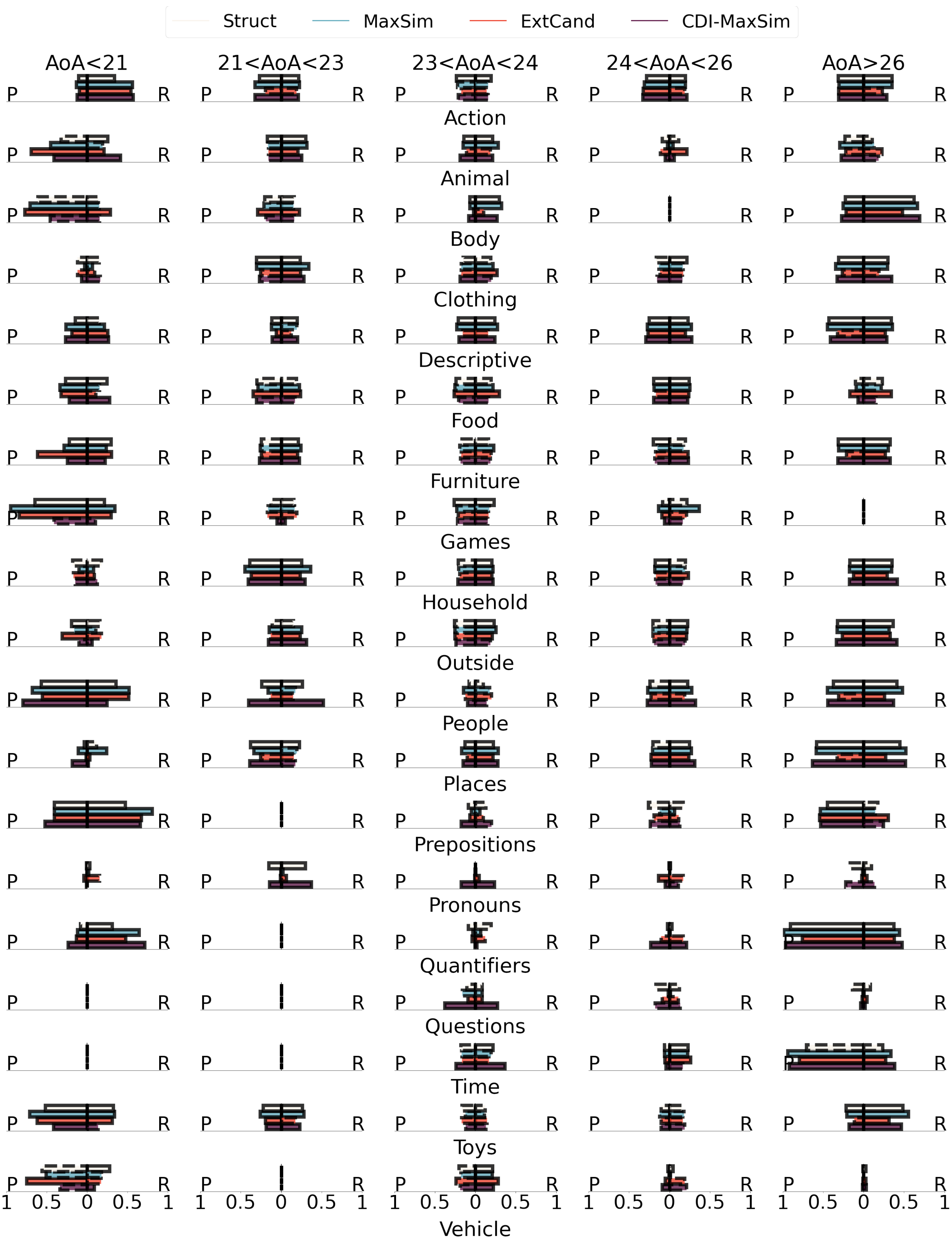

Figure 5: Precison-recall evaluation of all CDIs over all bins of age of acquisition.
